# A Rare Fracture

**Published:** 1879-08

**Authors:** Edward C. Huse

**Affiliations:** Rockford


### NOTES FROM PRIVATE PRACTICE.

### ARTICLE VIII.

*A Rare Fracture.*

On the night of the 5th of May, ult., Dr. F., of this city, æt. 27, called at my office, informing me that there was "something wrong" with his shoulder. He stated that upon getting out of bed an hour before, he had stumbled and fallen to the floor, striking his shoulder against something, which proved to be the edge of a door standing ajar. The pain was so excessive as to render him unconscious for some little time.

Upon careful examination of the part, I could at the outset detect no objective sign of injury. There was inability to place the hand upon the head, and extreme tenderness on pressure over a limited space just inside the acromial end of the clavicle and just below it. No crepitus or deformity was at this time observable. Accordingly I prescribed a hypnotic (morphiæ acetate 2 Ctgm.), and the application of a towel wrung out of hot water, on general principles. No opinion as to the lesion was expressed.

Next morning I called on the doctor, and at once noticed tumescence, circumscribed, at the injured spot, with considerable impairment of function, corresponding to the action of the pectoralis minor and coraco-brachial muscles. It will be remembered that the former is inserted into this process, and also that the conjoined tendon of the latter and of the short head of the biceps arise from its outer border. Crepitation was now quite perceptible. This was doubtless more from rupture of the trapezoid ligament than the separation of the fragments of bone. Indeed, when the relation of this ligament and also of the conoid are considered, it seems to me that this fracture implies the *necessary* rupture of both of them.

When we observe that the coracoid process is epiphyseal, and that complete osseous union does not occur till the twenty-fifth year or even later, this case may be viewed more as a separation than a fracture, perhaps. Hamilton, however, considers such separations under the nomenclature of fractures.

The exceeding infrequency of this injury is obvious from a consideration of the anatomical reasons. Nothing but direct violence of a special kind can cause it. I may add, and it is to be hoped, unassumingly, nothing but direct and close observation will detect it. Erichsen says only about a dozen cases of fractured coracoid are recorded. Mr. Lizars denies having seen a well authenticated case. Bransby Cooper and Dr. R. Mussey have each seen one. Hamilton has seen but one.

In conclusion, I would state that I had the honor to present this unique example, a few days after it occurred, before the Rockford Medical Association, and their opinion entirely concurred with my own as to the diagnosis. Hence the confidence with which I venture to assert the diagnosis.

The patient, it may be added, has but little inconvenience at present, no treatment further than elevation of the forearm upon the chest having been employed.

EDWARD C. HUSE, M.D.

ROCKFORD, June 2d, 1879.

---

THE Third Annual Session of the American Dermatological Association will be held in the rooms of the Academy of Medicine, No. 12 W. 31st Street, New York, on the 26th, 27th and 28th of August, 1879.

